# A Novel Algorithm for Determining the Contextual Characteristics of Movement Behaviors by Combining Accelerometer Features and Wireless Beacons: Development and Implementation

**DOI:** 10.2196/mhealth.8516

**Published:** 2018-04-20

**Authors:** Daniele Magistro, Salvatore Sessa, Andrew P Kingsnorth, Adam Loveday, Alessandro Simeone, Massimiliano Zecca, Dale W Esliger

**Affiliations:** ^1^ School of Sport, Exercise, and Health Sciences Loughborough University Loughborough United Kingdom; ^2^ National Centre for Sport and Exercise Medicine Loughborough United Kingdom; ^3^ International Center for Science and Engineering Programs School of Creative Science and Engineering, Faculty of Science and Engineering Waseda University Tokyo Japan; ^4^ Department of Mechatronic Engineering Faculty of Engineering Shantou University Guangdong China; ^5^ Wolfson School of Mechanical, Electrical and Manufacturing Engineering Loughborough University Loughborough United Kingdom

**Keywords:** context, indoor location, activity monitor, behavior, wearable sensor, beacons/proximity, algorithm, physical activity, sedentary behavior

## Abstract

**Background:**

Unfortunately, global efforts to promote “how much” physical activity people should be undertaking have been largely unsuccessful. Given the difficulty of achieving a sustained lifestyle behavior change, many scientists are reexamining their approaches. One such approach is to focus on understanding the context of the lifestyle behavior (ie, where, when, and with whom) with a view to identifying promising intervention targets.

**Objective:**

The aim of this study was to develop and implement an innovative algorithm to determine “where” physical activity occurs using proximity sensors coupled with a widely used physical activity monitor.

**Methods:**

A total of 19 Bluetooth beacons were placed in fixed locations within a multilevel, mixed-use building. In addition, 4 receiver-mode sensors were fitted to the wrists of a roving technician who moved throughout the building. The experiment was divided into 4 trials with different walking speeds and dwelling times. The data were analyzed using an original and innovative algorithm based on graph generation and Bayesian filters.

**Results:**

Linear regression models revealed significant correlations between beacon-derived location and ground-truth tracking time, with intraclass correlations suggesting a high goodness of fit (*R*^2^=.9780). The algorithm reliably predicted indoor location, and the robustness of the algorithm improved with a longer dwelling time (>100 s; error <10%, *R*^2^=.9775). Increased error was observed for transitions between areas due to the device sampling rate, currently limited to 0.1 Hz by the manufacturer.

**Conclusions:**

This study shows that our algorithm can accurately predict the location of an individual within an indoor environment. This novel implementation of “context sensing” will facilitate a wealth of new research questions on promoting healthy behavior change, the optimization of patient care, and efficient health care planning (eg, patient-clinician flow, patient-clinician interaction).

## Introduction

### Background

Contextual characteristics of the physical and built environment are known to affect health, both directly and indirectly, through the influence on individual activities and health-related behaviors [1-5]. Indeed, individual choices can be determined by social and physical environmental context [6], which may also affect the intervention strategies needed to influence and change behavior. Therefore, it is crucial to measure the time (the “when”), the place (the “where”), and the potential social settings where human movement behaviors (physical activity and sedentary time) occur. Recent methodological advances have emphasized the need for more holistic approaches, which can allow for greater specificity and flexibility in exploring and understanding the relationships between behavior and place [7,8]. Tracking and determining where specific movement behaviors are performed could provide valuable information and could greatly enhance research determining the correlates of physical activity and sedentary behaviors. Most commonly, movement behaviors (the “what”) are objectively measured using wearable devices such as accelerometers, which can record motion and postural changes over time [9]. However, there are limitations to accelerometery—most notably, their inability to accurately assess lifting and carrying, cycling, and water-based activities, and the general lack of contextual information relating to activity mode and/or location and domain [10,11]. Indeed, current methods of objectively assessing behavior do not provide information on the situational context of where behavior is conducted within free-living enclosed environments. Therefore, more appropriate technologies have been sought to measure the behavioral context. Improved measures would be of use in etiological studies in tracking trends in movement behavior within populations, making objective comparisons between populations, and monitoring the effect of interventions [12].

Global positioning systems (GPS) have been used to identify activities in outdoor locations [13,14]; however, a GPS cannot receive signals in the majority of indoor environments or provide room-level location [15]. This is problematic as most people spend the majority of their time in an indoor environment. Indoor tracking systems are a potential alternative solution [8], and according to the review of Loveday et al [16], several technological tools are available which are able to measure indoor location, for instance, Bluetooth low energy (BLE) beacons, radio-frequency identification, and real-time locating systems. These technologies have primarily been used to track activities in warehousing environments [17] or identify when a patient is in or out of their hospital bed [18]. Despite this, the technologies offer a great opportunity to be applied to the measurement of movement behaviors. BLE beacon functionality has also been incorporated within physical activity monitors, which provides the opportunity to assess duration, intensity, and context of movement behaviors only using one monitor. However, beacon functionality and support are limited, and there is currently a lack of validated algorithms that are using off-the-shelf activity monitoring products to aid behavioral scientists to determine location from the data. Validated and simple-to-implement algorithms for off-the-shelf activity monitors would increase the ability of behavioral scientists to utilize this innovative technology in their own research.

Furthermore, algorithms available in the literature use BLE and accelerometry for precise localization inside a specific room rather than multiple rooms or larger environments [14,19]. More accurate assessments of free-living behavior would help to (1) characterize the relationship between movement behaviors, (2) context and disease prevention (ie, the dose-response relationship), (3) assess the efficacy of intervention strategies, and (4) monitor the behavior and activity patterns of various populations [20].

### Objectives

The primary aim of this study was to conceive, create, and test a novel algorithm using accelerometry-based, proximity-enabled sensors to detect location for a more sensitive and accurate understanding of where specific movement behaviors are occurring in an indoor context. The secondary aim of this study was to provide a working example of how location and accelerometer data can be combined. By assessing these data together, a novel measurement and consequently a novel line of research can be created that is focused on the interactions of context and information about movement behaviors.

## Methods

### Experimental Protocol and Equipment

The algorithm was developed using data collected within a typical indoor workplace location at Loughborough University. The building comprised a multifloor, multiroom setting with a mixture of open-plan and partitioned office spaces.

As a location prediction may be influenced by individual factors such as walking speed and dwelling time in a given location, 4 trials were developed to model the potential effect upon indoor location acquisition:

- Trial 1: normal walking speed (self-paced at approximately 1.4 m/s), stopping for at least 3 min in each area (rooms and social areas) on a specified route.
- Trial 2: slow walking speed (self-paced relative to the normal speed walk at approximately 0.9 m/s or slower), stopping for at least 2 min in each selected area (rooms and social areas) on a specified route.
- Trial 3: fast walking speed (self-paced at approximately 2.0 m/s), stopping for at least 1 min in each area (rooms and social areas) on a specified route.
- Trial 4: the walking speed, dwelling time, and route were not prescribed (ie, not previously decided).

**Figure 1:**
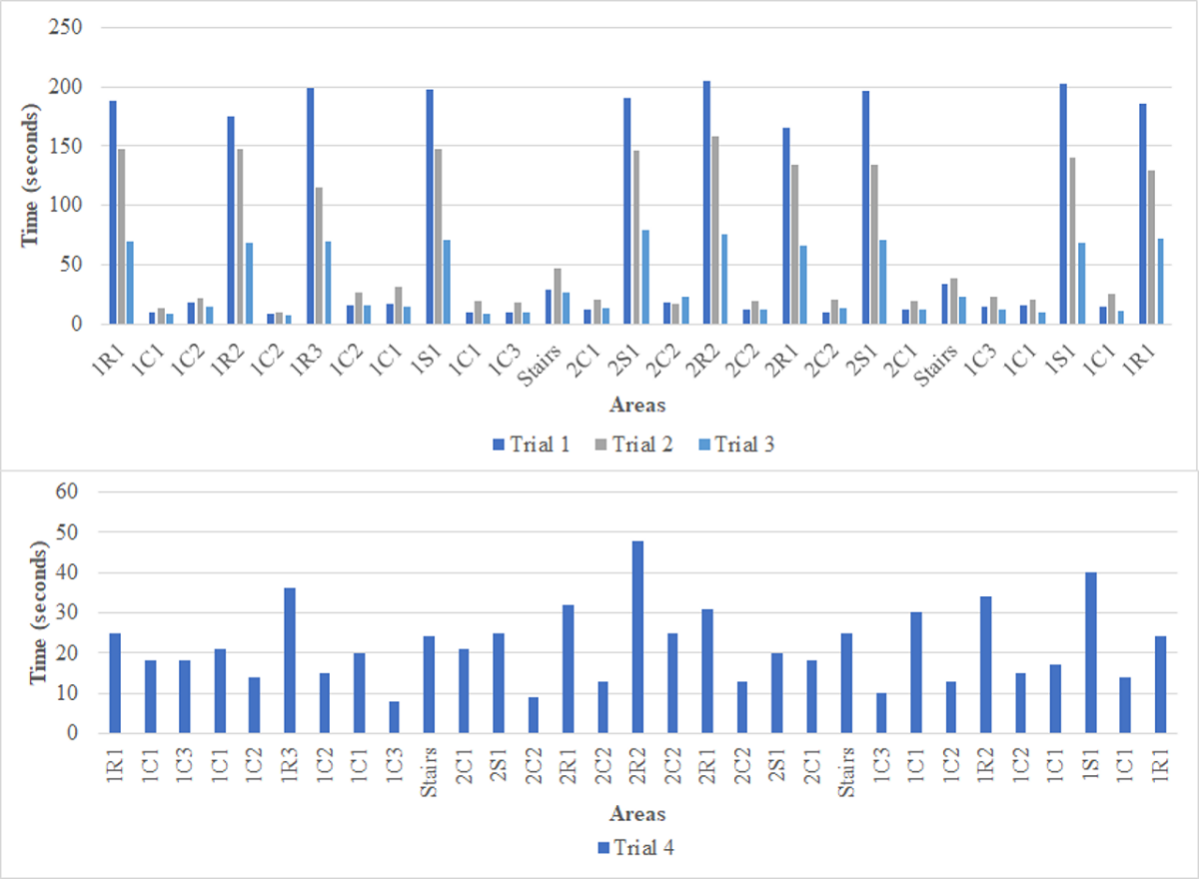
A visual representation, based on the areas order, of the dwelling time for each trial. Trial 1 at normal walking speed (self-paced at approximately 1.4 m/s); Trial 2 at slow walking speed (self-paced at approximately 0.9 m/s or slower); Trial 3 at fast walking speed (self-paced at approximately 2.0 m/s); Trial 4, the walking speed, dwelling time and route were not prescribed (ie, not previously decided). The locations are named as follows: First number indicates the floor: "1" indicates the first floor and "2" indicates the second floor; Uppercase letter indicates the type of room where the beacon was installed: "S" indicates a social area, "R" indicates a standard room, "C" indicates a corridor; Second number is a counter for the same type of room on the same floor; Lowercase letter is used only for long corridors or a large social area to indicate when multiple beacons were used; The label “Stairs” indicates the beacon placed in the stairway (same beacon on both floors).

Each trial started and finished in the same location, and all trials were also video-recorded using a wearable camera (HD-1080p, 60 fps) which served as criterion validity. A second technician recorded the sequence of the areas and the total time for each trial. Figure 1 represents the walking speed and dwelling time for each trial. Multimedia Appendix 1 represents the speed and dwelling time of each trial, and Multimedia Appendix 2 shows the path of trials 1 to 3.

ActiGraph accelerometers (GT9X Link, ActiGraph, Pensacola, United States) were used to provide time-stamped acceleration and indoor location. ActiGraph provides the most widely used accelerometers to measure physical activity and sedentary behavior. ActiGraphs are used within several national surveillance programs, including the US National Health and Nutrition Examination Survey. Researchers using ActiGraph in their own studies are therefore able to compare their own data with nationally representative samples. Equipped with BLE functionality, the devices can utilize proximity tagging, which allows for identification of other nearby devices. To enable location to be assessed, the devices are either initialized as a beacon or a receiver.

**Figure 2:**
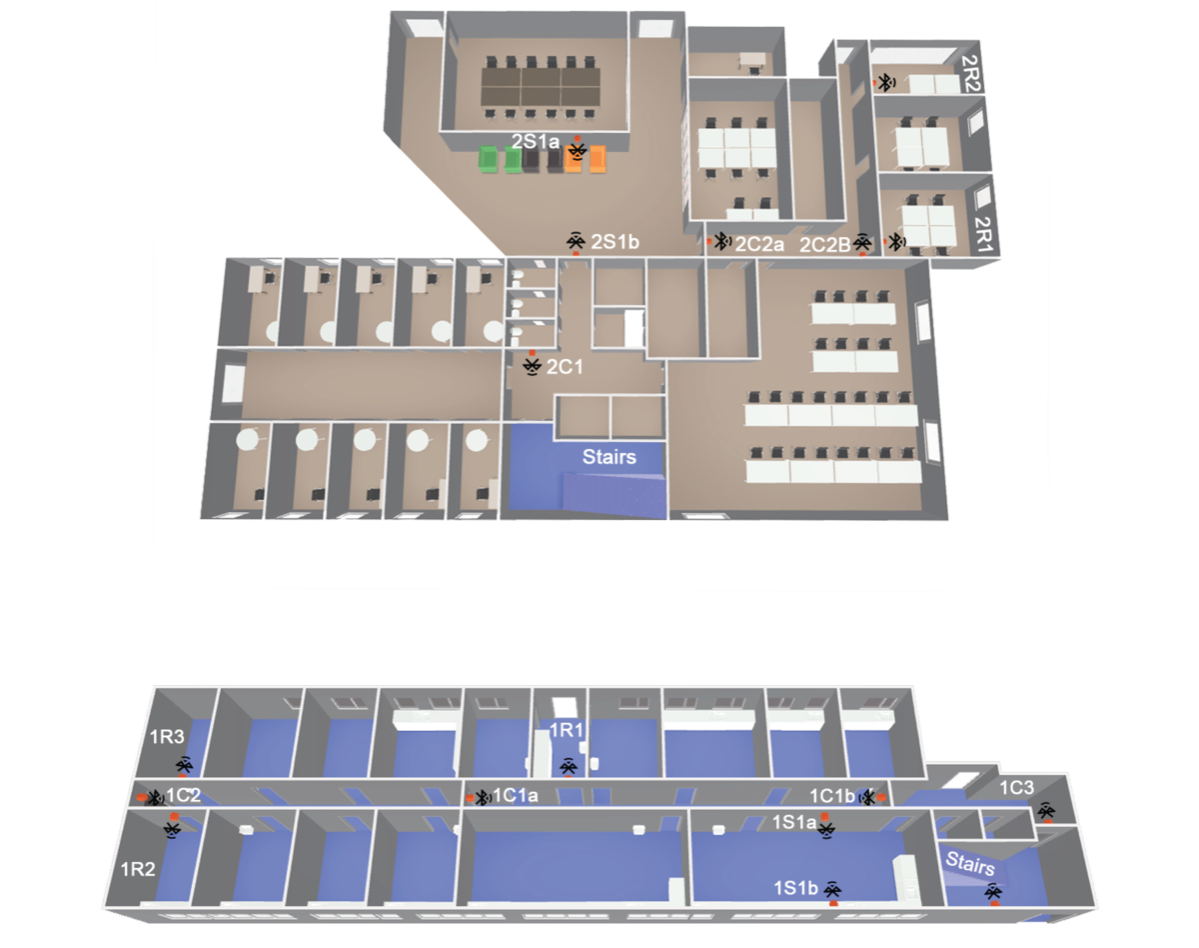
Building floorplan and beacon positions: Top: First floor; Bottom: Ground floor. Each beacon is shown in red with an accompanying Bluetooth symbol showing its direction.

Beacons should be placed around the environment in a high and unobstructed placement, if possible. Certain rooms were relatively small; therefore, one beacon was sufficient to cover the whole room and provide a discreet room occupancy measure (in line with ActiGraph recommendations). In these areas, the beacon was placed on the wall, 20 cm above the room’s door. To ensure sufficient social area coverage, more than one beacon was required. These were placed on 2 opposing walls of the area at 20 cm above the area’s door. Corridor beacons were placed in such a way that one beacon was used to cover a straight passage of a corridor (1 corridor required 2 beacons due to the length of the corridor), with a second beacon then placed when the corridor changed direction (always at 20 cm above the corridor’s door). The beacons were placed in this way to ensure reproducibility of the experimental situation in different built environments. To evaluate the performance of the proposed algorithm, 17 beacons were used in total, with 4 beacons used in 2 social areas (2 beacons in each), 1 beacon each in 5 rooms, 6 in corridors, and 1 in stairway (1 beacon). For a visual representation of the beacon locations, see Figure 2.

To track location, a receiver device is worn on the wrist, which records received signal strength indication (RSSI) readings from the beacons within the environment. In total, 4 ActiGraph devices were used as receivers, and 1 individual wore all devices, with 2 devices on each wrist. Receivers were initialized to record proximity at the highest sample rate possible (10-s intervals, 0.1Hz). Accelerometers were initialized and downloaded using ActiLife Version 6.13.3 (ActiGraph, Pensacola, United States).

In this study, accelerometers were initialized to collect acceleration data at a sample rate of 100 Hz. Accelerometer data were then processed to obtain a measure of activity every 10 s [21]. The metric selected is based on jerk (derivative of the acceleration) and consists of the following steps: (1) Module of acceleration:

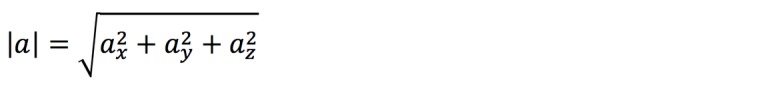



(2) moving SD of the module of the acceleration with window size 5 samples; and (3) average of the SD of the acceleration calculated in step 2 with nonoverlapping windows (window size 1000 samples, ie, 10 s at 100 Hz).

The first 2 steps aim to cancel the gravitational effect on the accelerometer data independently from the orientation of the sensor, while the third step defines the metric, which is proportional to the jerk level in the last 10 s. A k-mean classifier was then used to classify activity levels into low, middle, or high by using the previous metric from each test.

### Localization Algorithm

**Figure 3:**
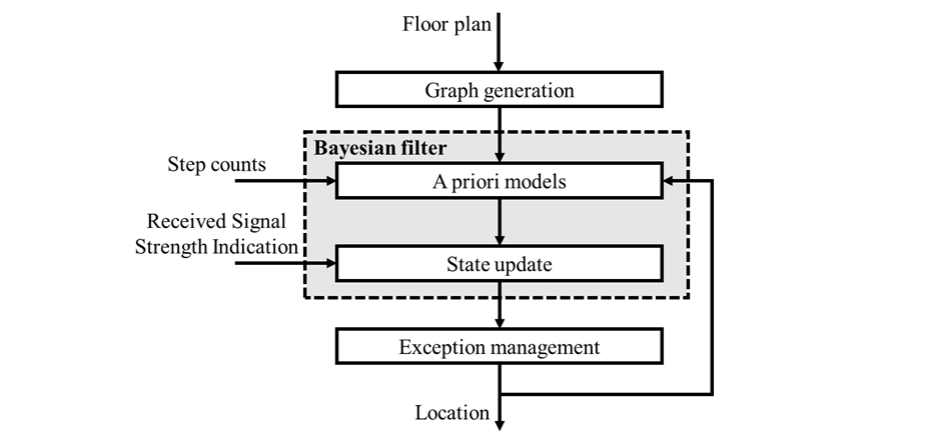
A schematic representation of how the localization algorithm was derived.

To produce the algorithm, a 4-step process is conducted that derives indoor location from receiver RSSI data: “graph generation,” “Bayesian filtering,” a priori motion model generation and state update, and “exception management.” This process is summarized in Figure 3.

#### Graph Generation

**Figure 4:**
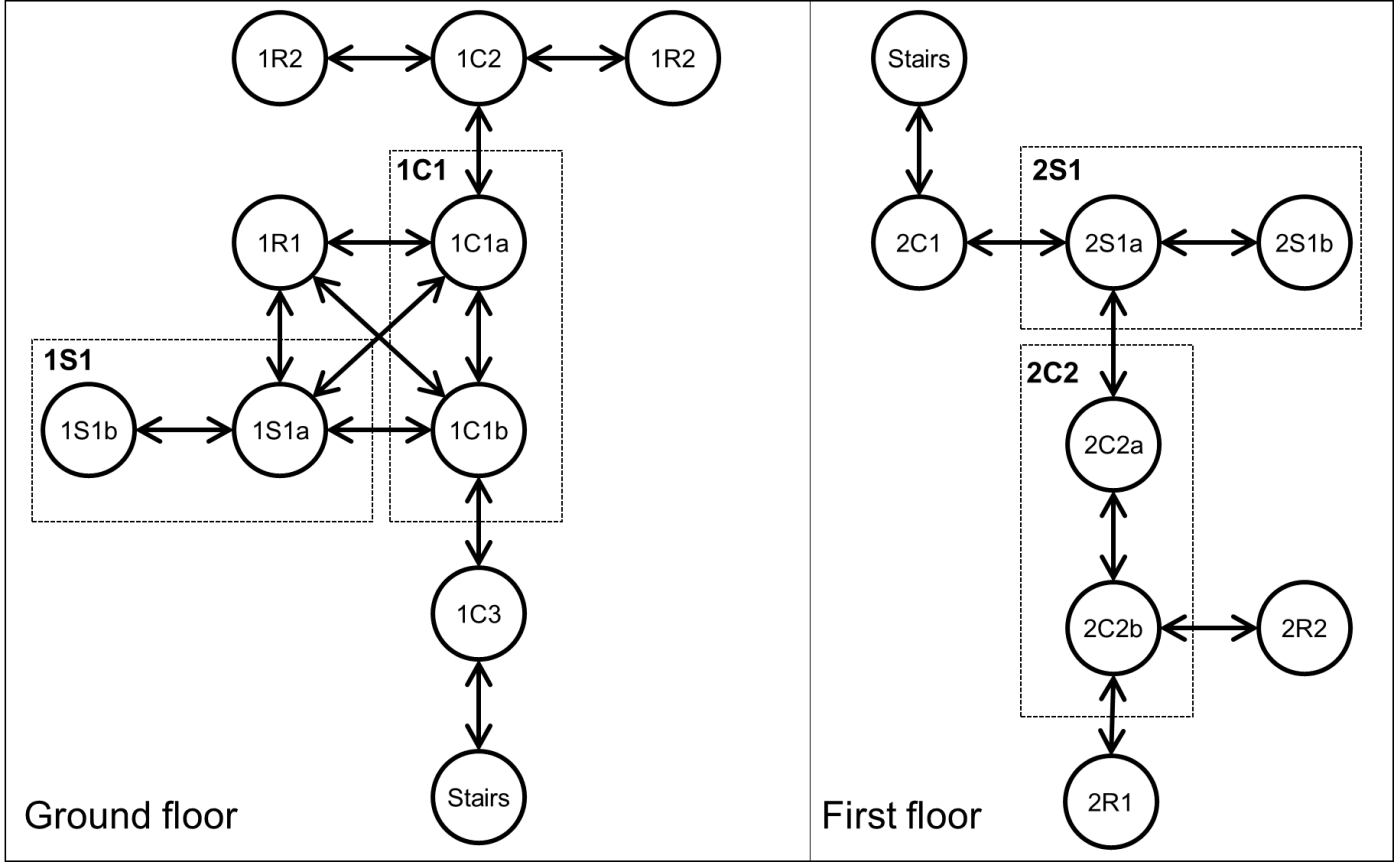
Map graph: the position of the beacons in the map transposed as a graph.

The position of the beacons on the floorplan is first represented as a graph, where each state represents the proximity to a specific beacon and the arrows indicate the possible paths among the beacons. Figure 4 shows the first and second floor graph used within the experiment; the first floor is connected to the second floor by the beacon “Stairs.” We grouped the beacons inside the same social area or corridor and named them excluding the lowercase letter.

The sparse transition matrix *A* represents the graph of the beacons:

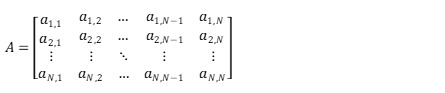



This matrix contains elements *a*_i,j_ that will assume the value:

1: if the beacons *i* and *j* are connected with a walking pathway,

0: if the beacons *i* and *j* are not connected with a walking pathway, and

*N*: number of beacons in the map.

i,j = 1 … N

#### Bayesian Filtering

Bayesian filtering aims to apply Bayesian statistics and Bayes rule to probabilistic inference problems [22]. It describes the probability of an event, in this case, the proximity to a beacon, based on prior knowledge of conditions that might be related to the event, such as the step counts and the proximity to a beacon in the previous sample. The criterion of optimality used for Bayesian filtering is the Bayes risk of minimum mean-square error [23]. Bayesian filtering is optimal because it searches for the posterior distribution, which integrates and uses all available information, in this case the RSSI measurement, expressed by probabilities [22]. Bayesian filtering works in 2 stages: (1) “a priori motion models” based where only the generated graph or step count measurements are used to estimate the probability of being in a specific room and (2) “state update” then corrects the estimation based on RSSI measurements.

##### A Priori Motion Models

In total, 3 a priori motion models were tested based on heuristic hypothesis on the movement of the participant.

###### Model 1

The connection weight among the beacons is constant, meaning that the technician can move to another room or stay in the same one with the same probability. This model can be described using a transition matrix model *M=A*.

###### Model 2

The connection weight among the beacons changes accordingly to the step count measurements. We can describe this model using a transition matrix model in which the elements *m*_i,j_ of the transition matrix model *M* are built as follows:

*m*_i,j_*= a*_i,j_ if *i ≠* *j*;

*m*_i,j_ = 0.5 if *i = j* and the step counts estimates more than 2 steps in the last 10 s (a higher number of steps indicates a lower probability of remaining in the same room); and

*m*_i,j_ = 2.0 if *i = j* and the step counts estimates 2 or less steps in the last 10 s (a lower number of steps indicates a higher probability of changing room).

###### Model 3

The connection weight among the beacons changes according to the step counts measurements but not for the corridors. This hypothesis is reasonable because the likelihood of a transition does not depend so strongly from the step counts measurements due to the length of a corridor. This model can be described using a transition matrix model in which the elements *m*_i,j_ of the transition matrix model *M* are built as follows:

*m*_i,j_*= a*_i,j_ if *i ≠* *j*;

If *i* is a beacon in a standard room or social area:

*m*_i,j_ = 0.5 if *i = j* and the step counts estimates more than 2 steps in the last 10 s;

*m*_i,j_ = 2.0 if *i = j* and the step counts estimates 2 or less steps in the last 10 s;

else

*m*_i,j_ = *w*

The value of *w* was manipulated in increments of 0.25 from 0.25 to 1.25. A lower value of *w* would indicate a higher likelihood to move into another room while in a corridor; in contrast, a higher value of *w* would indicate a lower likelihood to move in another room while in a corridor. If *w*=1.0, then the corridor was treated neutrally with respect to other transitions.

The position of the receiver can be defined as a state vector that contains the probability to be closer to a specific beacon. The position was estimated at the time *k*, based on the current state vector at the time *k* and the probability transition matrix as:

*X* ~_k + 1_ = *norm* (*X*_k_*M*)

where

*X*_k_ 1 × N state vector of the *N* probabilities (1 for each beacon) at the sample *k*;

*X* ~_k + 1_ 1 × N estimation of the *N* probabilities (1 for each beacon) at the sample *k* + 1; and

the state vector *X*_o_ is initialized with equal probability for all the beacons.

##### State Update

The probability of being in proximity to a beacon *i* at the sample time was heuristically estimated *k*, indicated as *y*_i_(*k*), based on the Bluetooth RSSI strength *RSSI*_i_:

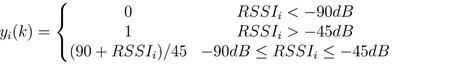



The rationale for these selections was:

1. The distance is inversely proportional to the RSSI in case of free air, although there are limitations on the estimation of distance indoor because of reflection and scattering [24].
2. The value of the RSSI does not exceed −45dB when the receiver is less than 1 m away from the beacon.
3. The communication is interrupted when the RSSI is less than −90dB.

The measurement vector was normalized at each sample time *Y*_k_ with respect to the sum of the probabilities of all the beacons:

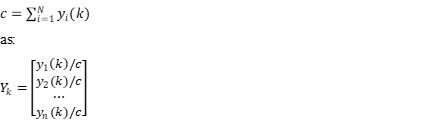



It is possible to measure the posterior probability *X*_k + 1_ based on *Y*_k + 1_ measurements and the estimation from the a priori model using the Bayes equation:

*X*_k + 1_ = *norm* (*X* ~_k + 1_ ⨀ *Y*_k + 1_)

where *X*_k + 1_ is the state vector of the *N* probabilities (one for each beacon) at the sample *k* + 1 and ⨀ indicates a point-wise vector multiplication.

The most probable position is the beacon *i*:

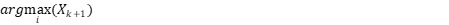



#### Exception Management

A block for exception management was implemented to make the algorithm more robust with respect to the following 3 failures:

1. This exception manages cases in which the estimated position based on the model is not in accordance with the RSSI measurements. Indeed, the measurement could not fit the estimated probability when there is at least one RSSI measurement (*c* ≠0) but the previous estimation is not fitting the a priori motion model (*X*_k + 1_ = 0 for all the beacons). This exception was managed by imposing a uniform probability for all the areas (resampling) and setting the updated state as the previous one because the RSSI measurement might have been corrupted by a glitch due to reflection or scattering.
2. There are no RSSI measurements (*c*=0), and therefore no update was performed in that step.
3. Due to reflection and scattering, the RSSI measurement could create hopping effects between 2 contiguous rooms. To improve the characteristic of stability in these cases, it was imposed that hopping from a room A to a room B and then back to the room A can happen only if the transition is stable for at least 2 samples (20 s). In this case, the updated probability of the room was left unchanged, and the most probable room before the hopping event was selected.

### Statistical Analysis

For the analyses, each transition period was defined as when the receiver moved from one room to the next. The total transition period for the trial *l* was indicated as *T*_t,l_. The total nontransition period for the trial *l* was defined as the remaining period and was indicated as *T*_nt,l_

The total time of the trial *T*_l_ was:

*T*_l_*= T*_t,l_ + *T*_nt,l_

We considered the errors on the transitions (*ê*_t,l_*)* and the errors on the nontransition (*ê*_nt,l_) for trial *l*, and we calculated the relative transition errors for trial *l*:

*e*_t,l_*= 100 ×* *ê*_t,l_*/ T*_t,l_

The relative nontransition errors for trial *l* was:

*e*_nt,l_*= 100 ×* *ê*_nt,l_*/ T*_nt,l_

The error caused by tracking problems on the transitions for trial *l* was:

*ē*_nt,l_*= 100 ×* *ê*_nt,l_*/ T*_l_

The relative error in trial *l* was:

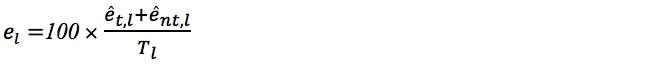



The average error on transitions was:

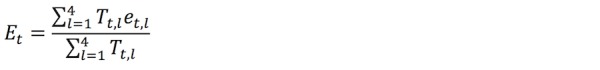



The average error on nontransitions was:

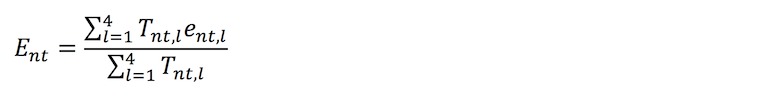



The average error caused by tracking problems on the transitions was:

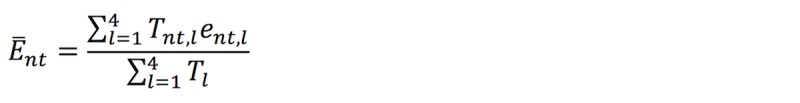



The average error was:

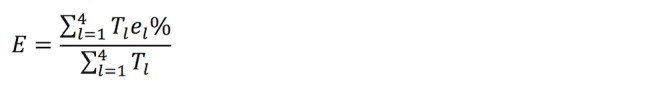



Furthermore, the total dwelling time within each room was evaluated for each trial to quantify the consistency of the location and videotaped tracking time. A two-way mixed intraclass correlation coefficients with absolute agreement was performed. Differences between estimates are reported as mean differences (95% CI). The regression model between the location tracking time and the videotaped tracking time was analyzed. The goodness of fit between the measured values and the expected value (videotaped tracking time) was tested by the following: sum of squares due to error (SSE), which is a measure of the discrepancy between the data and the model estimated *R*^2^, between 0 and 1, with a value closer to 1 indicating that a higher proportion of variance is accounted for by the model; adjusted *R*^2^, (ie, *R*^2^ adjusted for the residual degrees of freedom); and finally, root mean square error (RMSE, ie, the sample SD between predicted and observed values).

Accelerometry step counts were synchronized with the criterion video using timestamps from an exported .csv file, and the error was evaluated by measuring the time for which the estimated room position and the criterion were different. The validity of the algorithm was determined using the absolute percentage error scores calculated for each trial.

## Results

### Algorithm Performance

In general, all algorithm models calculated an average percentage error ranging from 14% to 17%. The two main sources of error within these models were (1) transition state, that is, moving from a room to a corridor or vice versa in the transition period and (2) nontransition state, defined as the dwelling time longer than 20 s (equal to two recorded samples of RSSI). Transition estimates improved when the algorithm used model 3 and the corridor was treated neutrally with respect to other transitions, where the connection weight among the beacons changed according to the step count measurements but not for the corridors (in which the weight was constant). The results for each model and trial are outlined in Table 1.

All algorithm models calculated low average error in the nontransition state with a decrease in error from 3% in the model 1 to between 0.31% and 0.47% in model 3 variations. In the transition state, an average error of between 31% and 34% was exhibited across all algorithm models. Generally, the relative error is affected more by transition errors if there is a higher transition rate.

**Figure 5:**
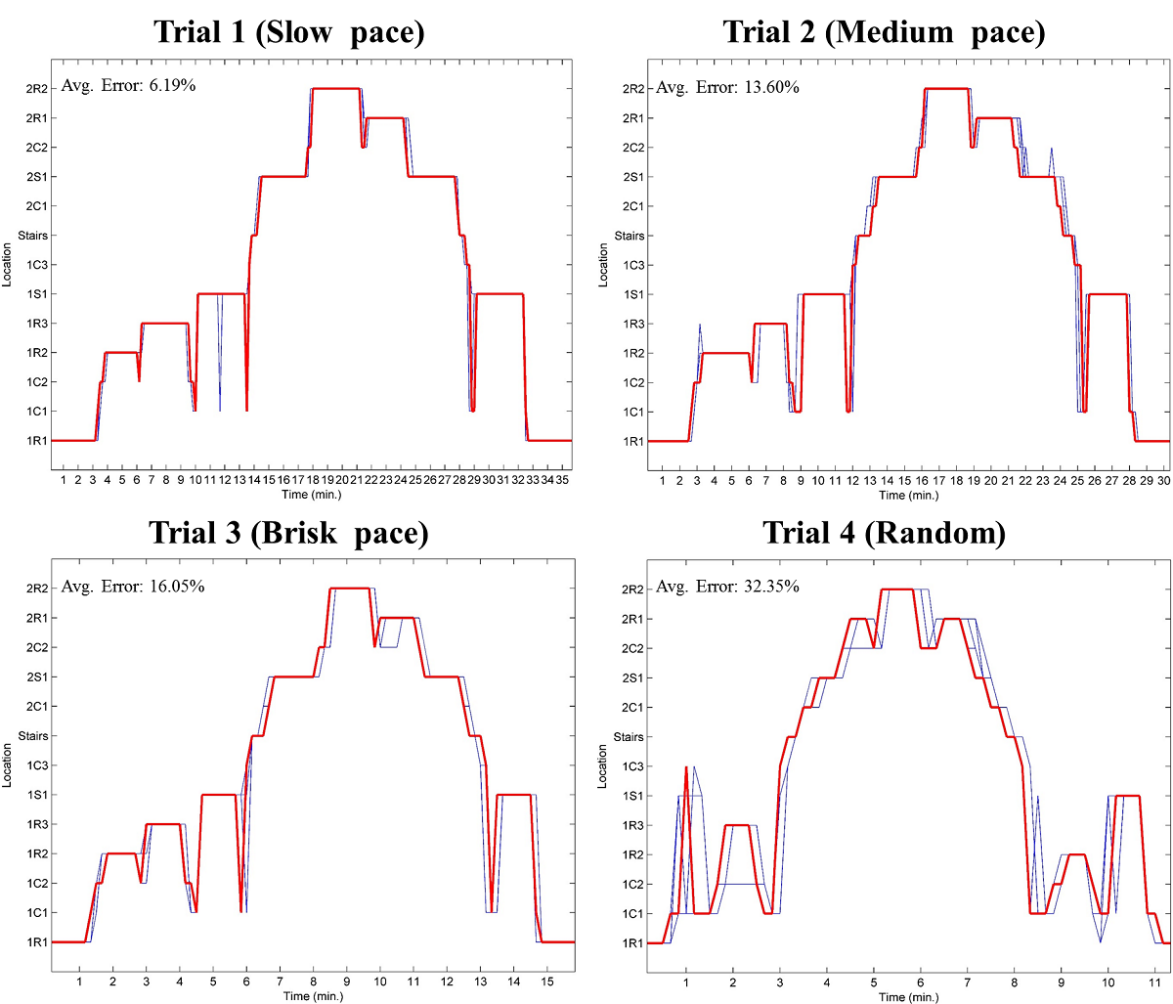
Tracking quality graphs for trials 1-4 (model 3; *w*=1.0). The red line represents location derived from the criterion measurement (camera), and the blue lines represent the locations obtained from the algorithm.

The tracking quality is represented by Figure 5 for all trials. The red line represents the criterion location obtained from the video, and the blue line represents the locations obtained from the 4 receivers. From these graphs, it is evident that the quality of tracking is better for trials with lower transition rates.

**Table 1:** Percentage error of transition and nontransition states for each trial and algorithm model. Italics is used to reflect the model that showed the best results. The letter *w* is the weight.

Algorithm model		Trial 1 (slow pace)	Trial 2 (medium pace)	Trial 3 (brisk pace)	Trial 4 (random)	Average error
**Model 1^a^, %**						
	Error on nontransitions *e*_nt,l_	3	8	6	8	6
	Error on transitions *e*_t,l_	27	40	25	3	32
	Error caused by tracking problems on the transitions *ē*_nt,l_	7	14	14	34	4
	Error *e*_l_	9	20	16	35	17
**Model 2^b^, %**						
	Error on nontransitions *e*_nt,l_	3	7		8	5
	Error on transitions *e*_t,l_	25	3	29	38	31
	Error caused by tracking problems on the transitions *ē*_nt,l_	6	13	16	35	14
	Error *e*_l_	8	17	17	35	16
**Model 3^c^** **(*w*=1.25), %**						
	Error on nontransitions *e*_nt,l_	0.31	4	2	8	3
	Error on transitions *e*_t,l_	23	39	31	38	31
	Error caused by tracking problems on the transitions *ē*_nt,l_	6	14	17	35	14
	Error *e*_l_	6	16	18	35	15
**Model 3 (*w*=1.00), %**						
	Error on nontransitions *e*_nt,l_	*0.31*	*1*	*2*	*4*	*1*
	Error on transitions *e*_t,l_	*25*	*39*	*30*	*35*	*31*
	Error caused by tracking problems on the transitions *ē*_nt,l_	*6*	*14*	*16*	*32*	*13*
	Error *e*_l_	*6*	*15*	*17*	*33*	*14*
**Model 3 (*w*=0.75), %**						
	Error on nontransitions *e*_nt,l_	0.31	1	2	4	1
	Error on transitions *e*_t,l_	25	39	31	34	32
	Error caused by tracking problems on the transitions *ē*_nt,l_	6	14	17	31	13
	Error *e*_l_	7	15	17	32	14
**Model 3 (*w*=0.50), %**						
	Error on nontransitions *e*_nt,l_	0.47	1	0	4	1
	Error on transitions *e*_t,l_	26	39	31	35	30
	Error caused by tracking problems on the transitions *ē*_nt,l_	7	14	17	32	14
	Error *e*_l_	7	15	17	32	14
**Model 3 (*w*=0.25), %**						
	Error on nontransitions *e*_nt,l_	0.31	1	0	4	1
	Error on transitions *e*_t,l_	27	41	34	36	34
	Error caused by tracking problems on the transitions *ē*_nt,l_	7	14	18	33	14
	Error *e*_l_	7	15	18	33	15

^a^Model 1: The connection weight among the beacons is constant.

^b^Model 2: The connection weight among the beacons changes according to the step count measurements.

^c^Model 3: The connection weight among the beacons changes according to the step counts measurements but not for the corridors.

**Figure 6:**
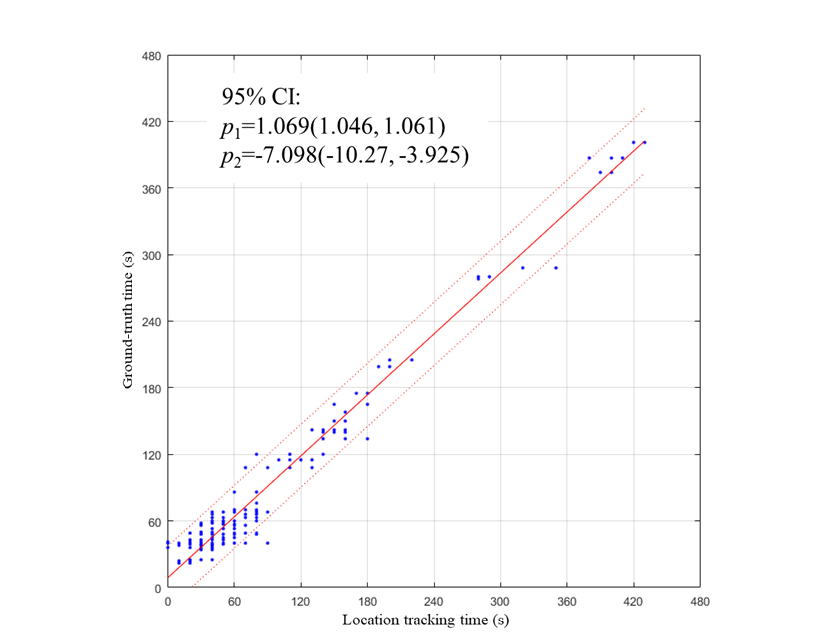
Linear regression model between accelerometry and criterion measure (video) tracking time in seconds.

Linear regression models *y = p*_1_*x + p*_2_ showed coefficients with 95% CI of *p*_1_=1.069 (1.046-1.061) and *p*_2_=−7.098 (−10.27 to −3.925) between algorithm and criterion time in seconds (Figure 6). Moreover, the intraclass correlation highlighted a very high goodness of fit of the model *R*^2^=.9780 (Table 2).

Greater location prediction robustness was obtained when the receivers spent over 100 s within a specific area, which is equivalent to more than 10 samples (quantization error less than 10%), for which we obtained an *R*^2^=.9775 (Table 2). Conversely, the algorithm poorly estimates dwelling time (*R*^2^=.4719) when the time spent in an area is under 100 s (Table 2).

RMSE is between 15.28 s and 15.62 s (see Table 2) independent from the time spent in a specific area. For this reason, the main source of error can be attributed to the transitioning of the receiver from one area to another because:

1. the highest amount of error is observed during transitions (3%-34%; Table 1); and
2. the sampling rate of the sensor is 10 s. The sampling effect is evident in the gaps between the estimations in Figure 5.

**Figure 7:**
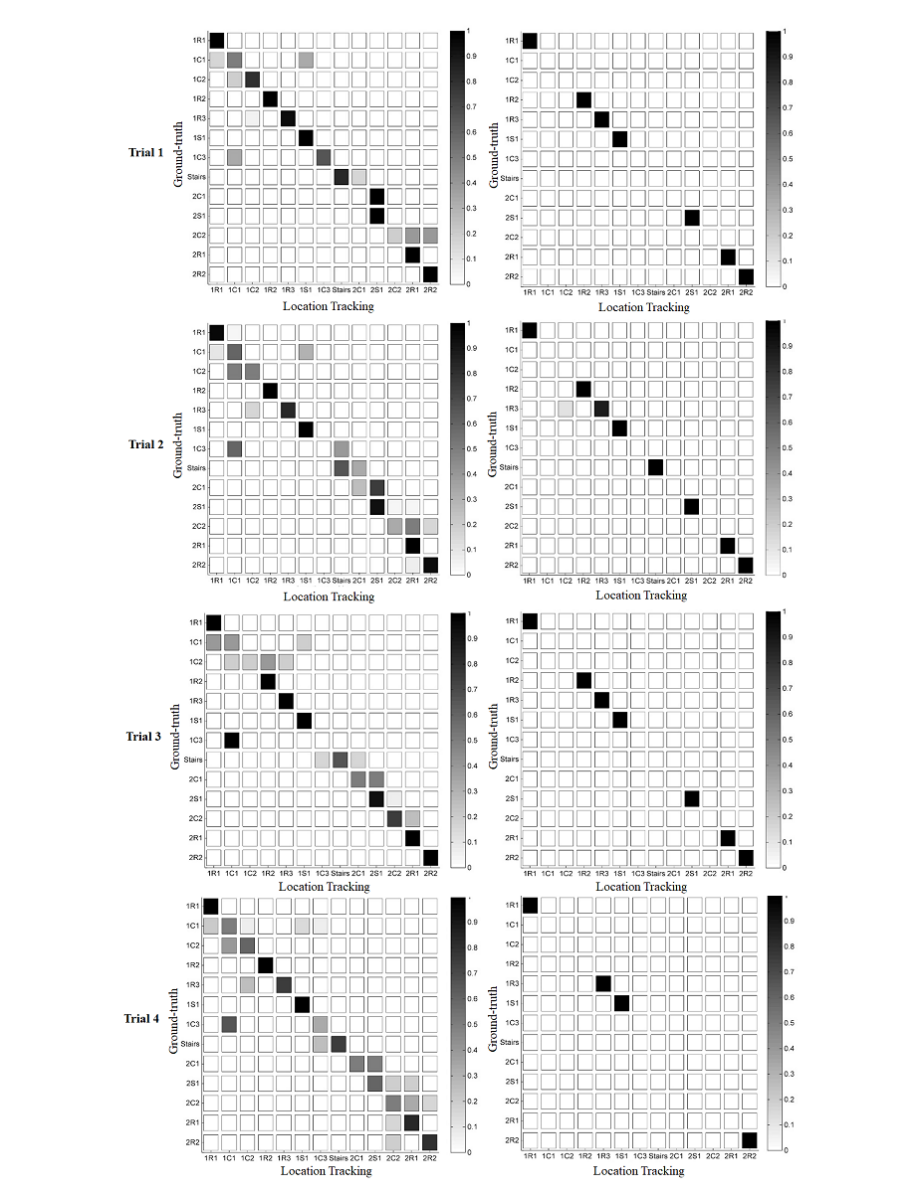
Confusion matrices of model 3 (*w*=1.0) for trials 1-4. Column A represents all the transition and nontransition states, and B only nontransition states.

Column A of Figure 7 displays the confusion matrices of model 3 (*w*=1.0) for all trials. A darker diagonal line represents a better prediction from the algorithm. From the graphs, it is evident that errors often occur between adjacent beacons. For trial 1 (column A), the algorithm confused 1C1 with 1S1 because beacon 1C1b is close to 1S1a. Likewise, for trial 3 (column A), the algorithm confused 1C1 with 1C3 because beacon 1C1b is close to 1C3. The close proximity of certain beacons increases the possibility that refraction effects lead to a misclassification of the room that the receiver is located within. However, this effect is only evident in the case of transitions, and it never happens for beacons placed on different floors even though the distance between the beacons is relatively small, such as in the case of the beacons 2C1 and 1C3.

Column B displays the confusion matrices of model 3 (*w*=1.0) for all trials but only in nontransition states. Location prediction was high (values higher than 0.99) within nontransition states such as the standard rooms (R), the social areas (S), and the stairs. However, within trial 2, the algorithm confused the standard room 1R3 and the corridor 1C2 in 10% of the cases because of the proximity between the beacons. Therefore, precautions must be taken in the placement of the sensors to avoid such ambiguities.

**Table 2:** The goodness of fit between the measured and the expected values of model 3 (*w*=1.0). SSE: sum of squares due to error, which is a measure of the discrepancy between the data and the model estimated; RMSE: root mean square error that is the standard error of a measurement, the standard error of the regression.

Measurements	SSE (s^2)	R-squared^a^	Adjusted R-squared^b^	RMSE (s)
Measurements under 100 s (130 points)	31,700	.4759	.4719	15.62
Measurements over 100 s (73 points)	17,290	.9775	.9772	15.28
All measurements (203 points)	49,543	.9780	.9779	15.51

^a^*R*^2^, between 0 and 1, with a value closer to 1 indicating that a higher proportion of variance is accounted for by the model.

^b^Adjusted *R*^2^ that adjusts *R*^2^ for the residual degrees of freedom.

**Figure 8:**
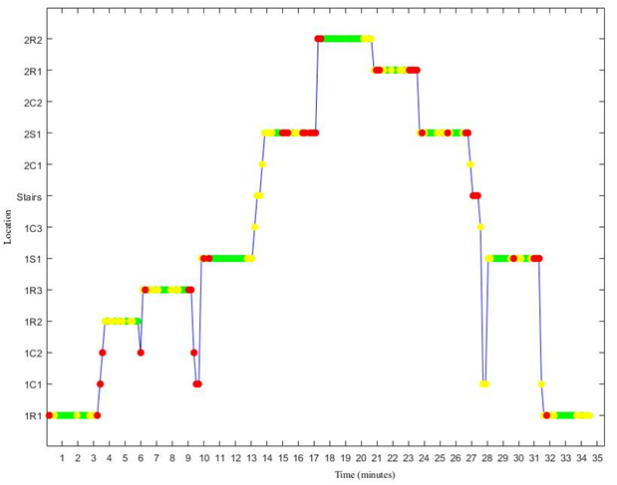
The combination of localization tracking and activity data of trial 1. The green, yellow, and red color indicate low, middle, and high level of activity, respectively.

### Behavioral Analysis

Given that the proposed algorithm is able to accurately determine indoor location, it is worth considering the utility this offers researchers in assessing where behavior occurs indoors. Figure 8 shows the combination of localization tracking and activity data in the case of trial 1. It is evident that the highest levels of activities are conducted between rooms, in corridors and stairs, just before and after transition. Conversely, activities of a low or moderate nature were most commonly conducted within rooms.

**Figure 9:**
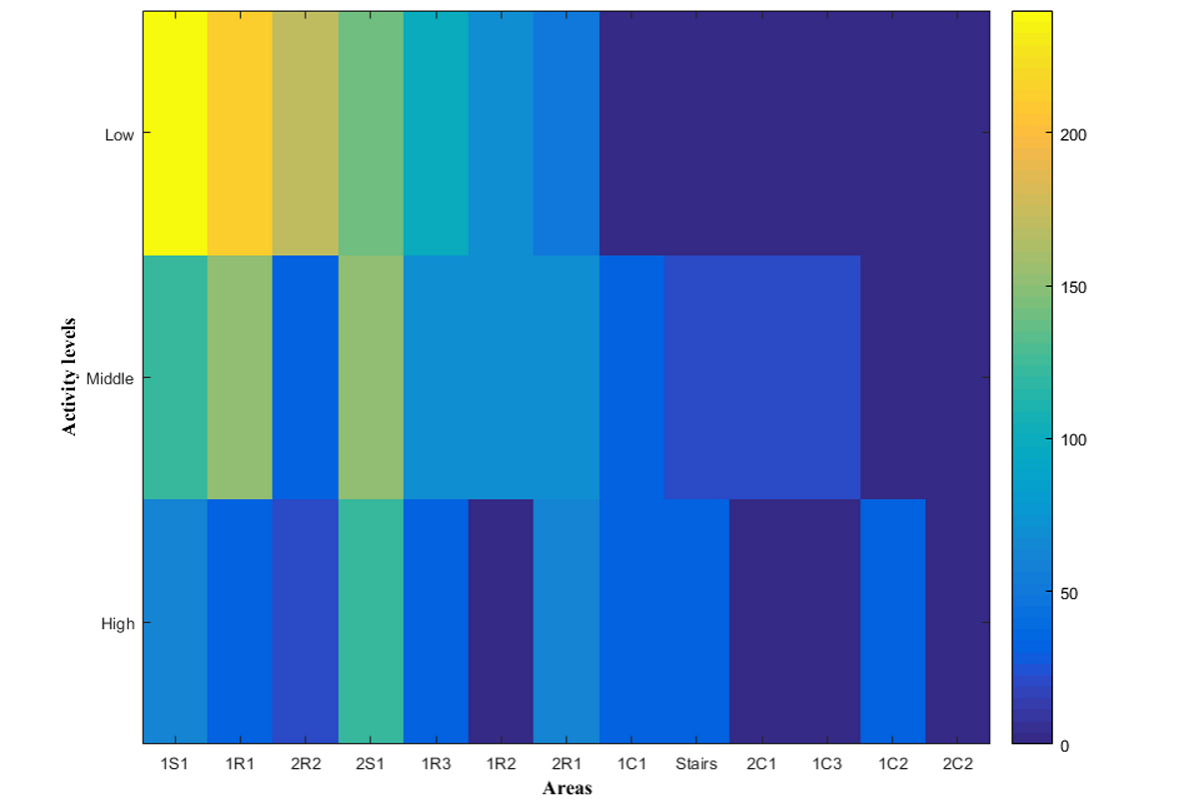
Lasagna plots representing the combination of localization tracking and activity levels. Areas are sorted in descending order depending on the absolute time. The color between blue (time equal to zero) and yellow (time equal to 230 s) represents the activity level spent-time.

The combination of localization tracking and activity levels can be used to create a Lasagna plot. The absolute time associated to each room and activity level is represented by a color gradient from blue (time equal to 0) to yellow (time equal to 230 s). The graphs were initially produced without sorting the data by absolute time, and this made it difficult to extract useful information. Therefore, for Figure 9 (trial 1), locations were sorted in descending order based upon absolute time. It is clear that there are areas where the receiver spent longer periods of time such as within rooms and social areas and areas where the time spent was minimal such as within corridors and stairs. Multimedia Appendix 3 represents the combination of localization tracking and activity levels with the area sorted based on the route of trial 1.

**Figure 10:**
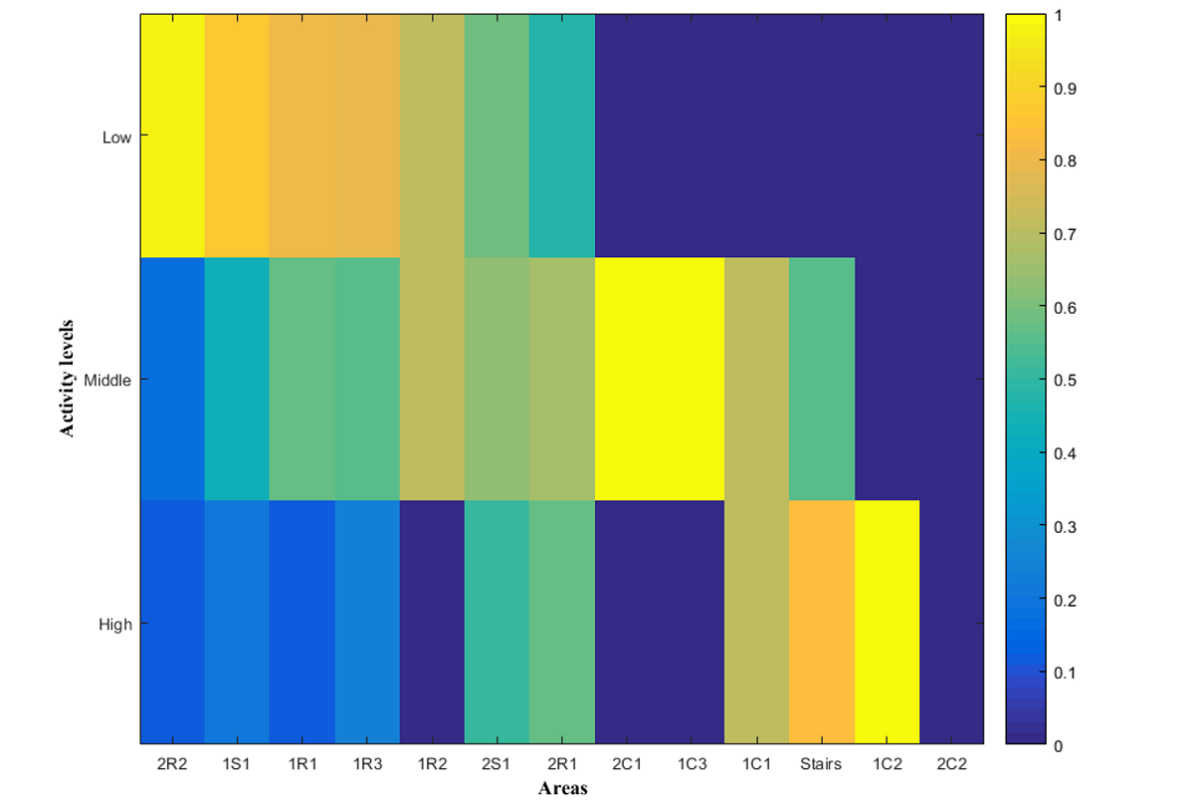
Lasagna plots representing the combination of localization tracking and activity levels. Areas are sorted in descending order depending on the relative time. The color between blue (time equal to zero) and yellow (time equal to 230 s) represents the activity level spent-time.

We also considered the relative time to compare the activity levels in each room regardless of the time the participant stays in a specific area. Figure 10 represents sorted location data based upon the relative time at low-level activity. The rooms and social areas are mainly represented on the left side of the plot (indicating predominantly low activity in these areas), while the corridors and stairs on the right-hand side (indicating predominantly high activity in these areas). Multimedia Appendix 4 represents the combination of localization tracking and activity levels with the area sorted based on the route of trial 1.

## Discussion

Understanding the contribution of context to the health-behavior relationship first requires the accurate measurement of location, which is often overlooked when quantifying movement behaviors. In this study, an algorithm was presented that integrated indoor location and accelerometer measured step counts to create a better representation of the human-environment interactions. This approach to indoor location facilitates the assessment of 3 main elements: (1) time, (2) location, and (3) activity level. This is advantageous over deriving time and activity levels alone, as discerning the contribution of context to movement behaviors will allow researchers to create location-specific interventions that may act as more potent “levers” for sustained lifestyle behavior change.

### Algorithm Performance

Generally, the algorithm models calculated lower error during nontransition states, which could be explained by the fact that the time of each transition was always faster than the sampling rate of the sensors (see Table 2). Consequently, the transition state error is always higher, where the location time was shorter. This is not related to the time per se but the RSSI recording as the signal may be collected in the previous or next location during the sample interval of 10 s. Transitional error is high probably using the current methodology and contributes approximately 30% of error within the models. Sampling rate is currently manufacturer limited; however, an investigation into higher sampling rates should be explored to ascertain if error associated with transitional states can be reduced, or if specific guidance can be provided to account for the RSSI transition. Moreover, a potential way to improve the performance of the algorithm could be by modeling the RSSI distribution [14,19], but this kind of static model is not practically applicable in realistic environments due to dynamic changes in RSSI brought about by refraction and scattering. In this study, we endeavored to test the algorithm in a realistic environment, where it is more likely there will be a nontransitional situation (ie, dwelling in 1 location) than transitional (ie, not making frequent transitions between areas).

### Context and Activity

Considering the combination of accelerometric and location data within specific built environments makes it possible to objectively assess the contribution of different environments to overall levels of behavior [7]. This type of data can answer questions about environmental exposure and behaviors that are specific in particular locations or at certain times of day. Typically, accelerometers provide objective information on physical activity intensity and duration. While the intensity of the activity may be an important concept for health, it lacks specificity to recognize specific activities [10]. The addition of context to accelerometer data provides an opportunity to develop a novel line of research focused on the interactions of environment and movement behaviors in a temporal, spatial, and behavioral way [7]. Moreover, intervention studies that measure changes in locations and behavior will provide more robust evidence of the effects of the interventions and their causality [25].

Typically, to record and analyze location and accelerometer data, different devices are used, which requires the synchronization of multiple data sources. This can be challenging; in fact, poor synchronization could directly impact the proper processing and interpretation of the data. In this study, only one sensor was used to record both measures. This is an important and, to our knowledge, novel step in indoor location research. The timestamped data from the ActiGraph receivers can give a rich and detailed sequence of the types of activities people are engaging in (intensity, duration, etc), what spaces are used, and at what time in the day. In fact, as time-based analysis is further integrated into behavioral studies, more complex space-time modeling analysis and strategies can be applied to model the possible range of activities that an individual can engage in, and to determine if events and exposures during one day could influence activities during the next day [7]. A prerequisite for this level of space-time modeling is an accurate measure of time spent in a given location. Additionally, location data could help researchers better understand if spending more time in one environment influences certain behaviors, if there is a specific number of times a person must be in an environment to exhibit certain behaviors, or if there are specific times of the day to observe certain behaviors. Subsequently, the introduction of the additional time and behavioral variables could offer a novel insight into locations that are promoting or inhibiting of a behavior.

The combination of location data with behavior is a starting point for researchers to understand the determinants of different behaviors in indoor environments. For example, the illustrative combination of accelerometer and location data from this study showed that more activity was performed during the transitions from one area to another than within certain rooms or social areas. Furthermore, it was possible to identify a third cluster of areas (2S1-IC3) in which the participant performs mostly middle-level activities. From this, behavior could be classified as mainly low, middle, or high, potentially giving insight on where the participant mainly performs their daily activities.

To the best of the authors’ knowledge, the approach implemented herein is novel. In particular, being able to provide objectively measured data regarding changes in location and activities, rather than self-reported information, may provide greater utility and relevance to researchers and stakeholders. Simple epidemiological measures of behavior and activity, such as questionnaires, have performed adequately to demonstrate associations with a number of chronic disease outcomes; however, they rarely separate activity into its different dimensions, nor have they facilitated an estimation of dose-response effects [12]. Therefore, improved measurement methods, such as indoor location tracking, would be of use in epidemiological studies to record trends in behavior and activity within populations, making objective comparisons between populations, and in monitoring the effect of interventions and programs [12]. Nevertheless, due to the lack of research within the contextual domain, it is still unclear what additional benefit will be added to our current understanding. It is therefore essential that algorithms such as the one presented within this paper be refined and used in practice before the quantification of context is advocated as a necessary measurement within behavioral research.

### Limitations

Some technical and practical difficulties associated with the approach reported within this paper must be highlighted. First, one issue relates to difficulties in establishing a valid protocol that can ensure consistent performance in different environments, without the need for ad hoc testing and calibration. In fact, some aspects are likely to deviate to some degree, such as the possible variation in performance coming from differences in building materials and morphology.

Quantifying contextual movement behaviors using the presented technologies could be considered costly due to the number of devices required to accurately capture location over multiple domains. These limitations equally apply to existing measurement practices such as the combination of accelerometry and GPS [7]; however, these practices do not appear to have been decisively held back by these limitations. This is, presumably, due to the idea that the value added by the combination of activity data and GPS outweighs these limitations [7]. Future iterations of contextual technologies will hopefully alleviate these concerns.

A final limitation is related to the contextual tracking system that was used. The ActiGraph model does not allow the user to adjust the strength of the BLE signal or the sampling rate for data collection. In our opinion, the possibility to adapt the signal of the beacons to the size of the different areas could improve the performance of the tracking location algorithm. Indeed, this could avoid situations where signals from beacons in multiple rooms are recorded simultaneously. Additionally, the maximum sampling rate for the BLE signal is 10 s. There are situations where transitioning between 2 or more adjacent areas could take place between samples and consequently, the algorithm would not be able to accurately track location. A higher sample rate of the BLE signal, for example, 5 s or even every second, would make it possible to have a more precise indoor tracking output.

### Conclusions

The aim of this study was to create and test a novel algorithm, using accelerometry-based proximity-enabled sensors, to combine the “where,” the “when,” and the “what” of movement behaviors by the novel exploitation of existing technologies (conventionally used only to understand the “what”). Compared with a criterion measure, it has been demonstrated that the new approach can reliability predict location within rooms and social areas; however, there is increased error for transition between areas. This combination allows us to objectively and reliably determine the individual characteristics of contextual behavior. This new information can be used to better inform evidence-based practice and research interventions. The results have shown that it is possible to capture location information in indoor environments and that this can be combined with activity monitoring data to create variables previously unavailable for research.

It is clear that many behaviors and health issues are directly related to the context; thus, this novel approach is a powerful tool for researchers to monitor the “where,” “when,” and “what” of daily activities. As a first step into utilizing both context and behavior from one device, there is a need to conduct more research to refine the algorithm and bring about more technological advancement to reduce the current limitations, before indoor location can be utilized within intervention and epidemiological research.

Our study shows that a novel implementation of “context sensing” will facilitate a wealth of new research questions on promoting healthy behavior change, the optimization of patient care, and efficient health care planning (eg, patient-clinician flow; patient-clinician interaction). This fresh perspective will help both researchers to develop new strategies to study human behavior, and policy makers to design new public health initiatives aimed at improving positive and functional behavior within the population.

## Appendix

Multimedia Appendix 1 A visual representation of the speed and dwelling time of each trial.

Multimedia Appendix 2 A two-dimensional floorplan representing the beacon locations and path of trials 1-3.

Multimedia Appendix 3 Lasagna plots representing the combination of localization tracking and activity levels. Areas are not sorted by absolute time. The color between blue (time equal to zero) and yellow (time equal to 230 s) represents the time being active.

Multimedia Appendix 4 Lasagna plots representing the combination of localization tracking and activity levels. Areas are not sorted in descending order by relative time. The color between blue (time equal to zero) and yellow (time equal to 230 s) represents the activity level spent-time.

## Abbreviations

BLE: Bluetooth low energy
GPS: global positioning system
RMSE: root mean square error
RSSI: records received signal strength indication
SSE: sum of squares due to error
