# Supplementary figures and images for: A Novel Algorithm for Determining the Contextual Characteristics of Movement Behaviors by Combining Accelerometer Features and Wireless Beacons: Development and Implementation
Source: JMIR Mhealth Uhealth. 2018 Apr 20;6(4):e100. doi: 10.2196/mhealth.8516 (PMC5935802; doi:10.2196/mhealth.8516)

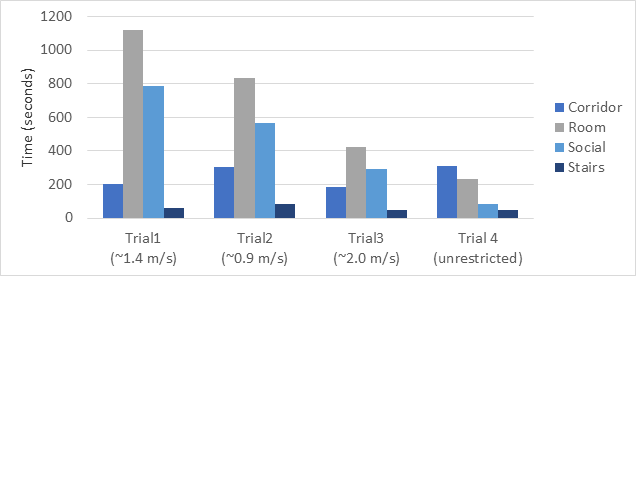

Supplement: Multimedia Appendix 1 [file mhealth_v6i4e100_app1.png]

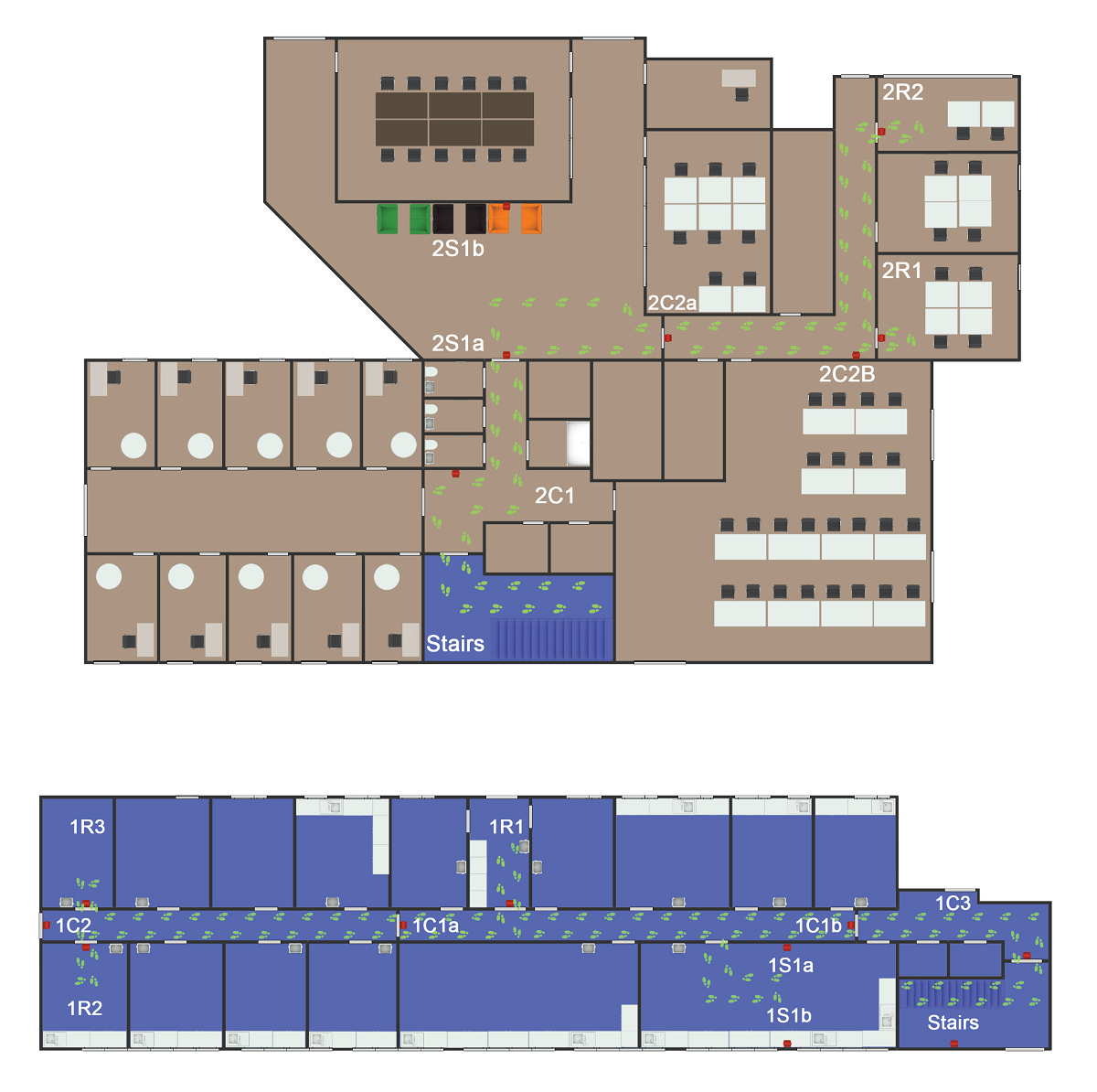

Supplement: Multimedia Appendix 2 [file mhealth_v6i4e100_app2.png]

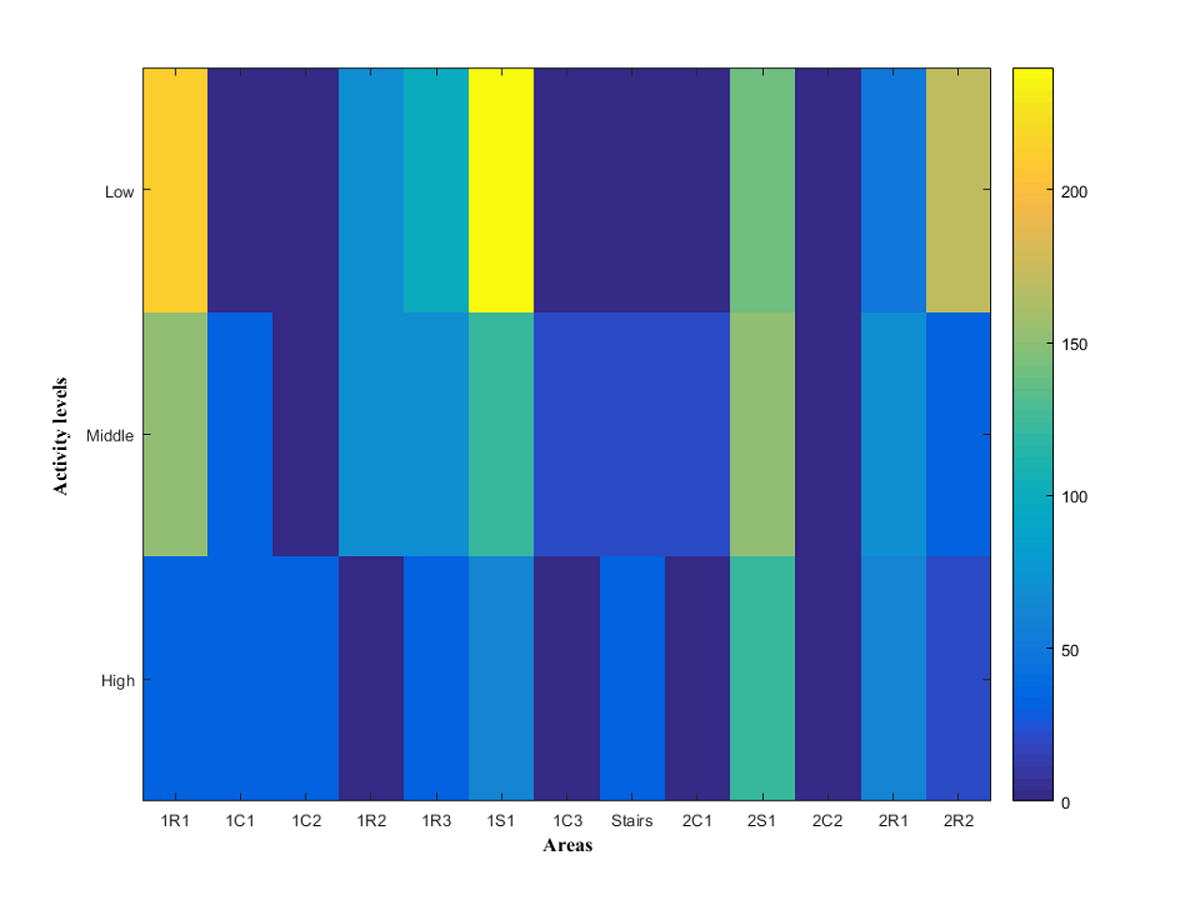

Supplement: Multimedia Appendix 3 [file mhealth_v6i4e100_app3.png]

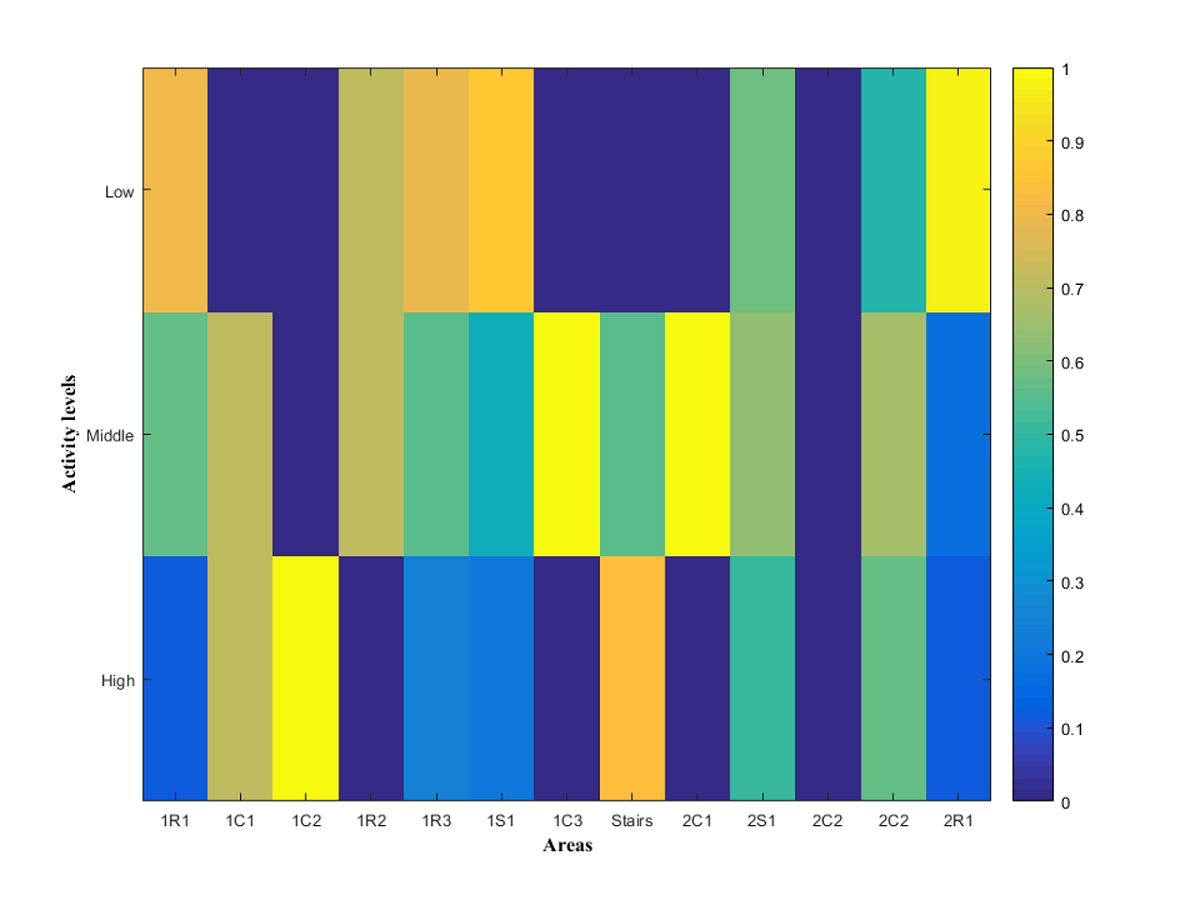

Supplement: Multimedia Appendix 4 [file mhealth_v6i4e100_app4.png]
